# Supplementary figures and images for: Functional morphology of the Cambrian archaeocyath sponge Yukonensis
Source: PLoS One. 2026 May 11;21(5):e0347476. doi: 10.1371/journal.pone.0347476 (PMC13160350; doi:10.1371/journal.pone.0347476)

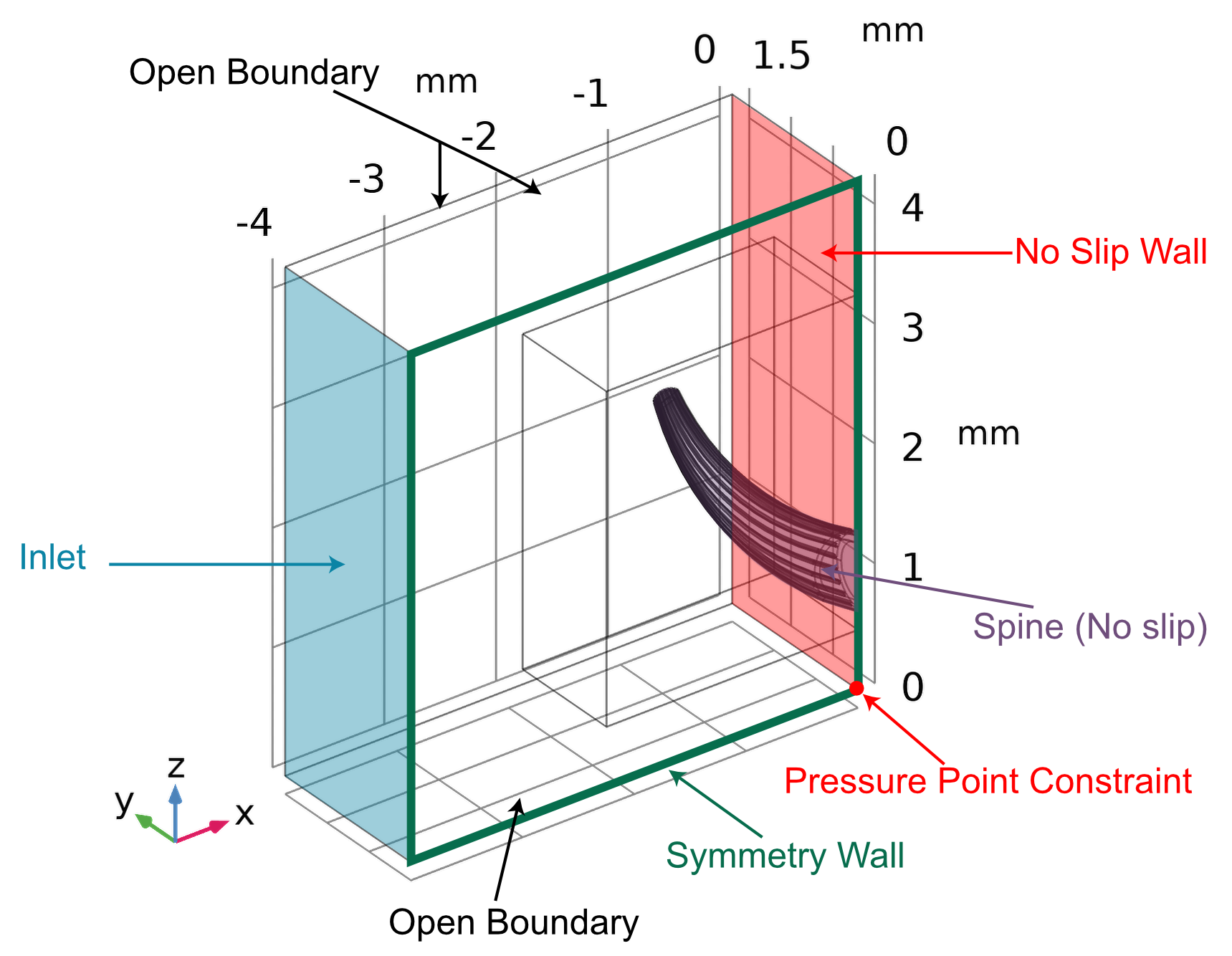

Supplement: S1 Fig — Wall colour corresponds to the colour of the text. The symmetry wall is highlighted by a dark green outline. The pressure point constraint is visualized by the red dot on the symmetry wall’s bottom right corner. (TIF) [file pone.0347476.s004.tif]

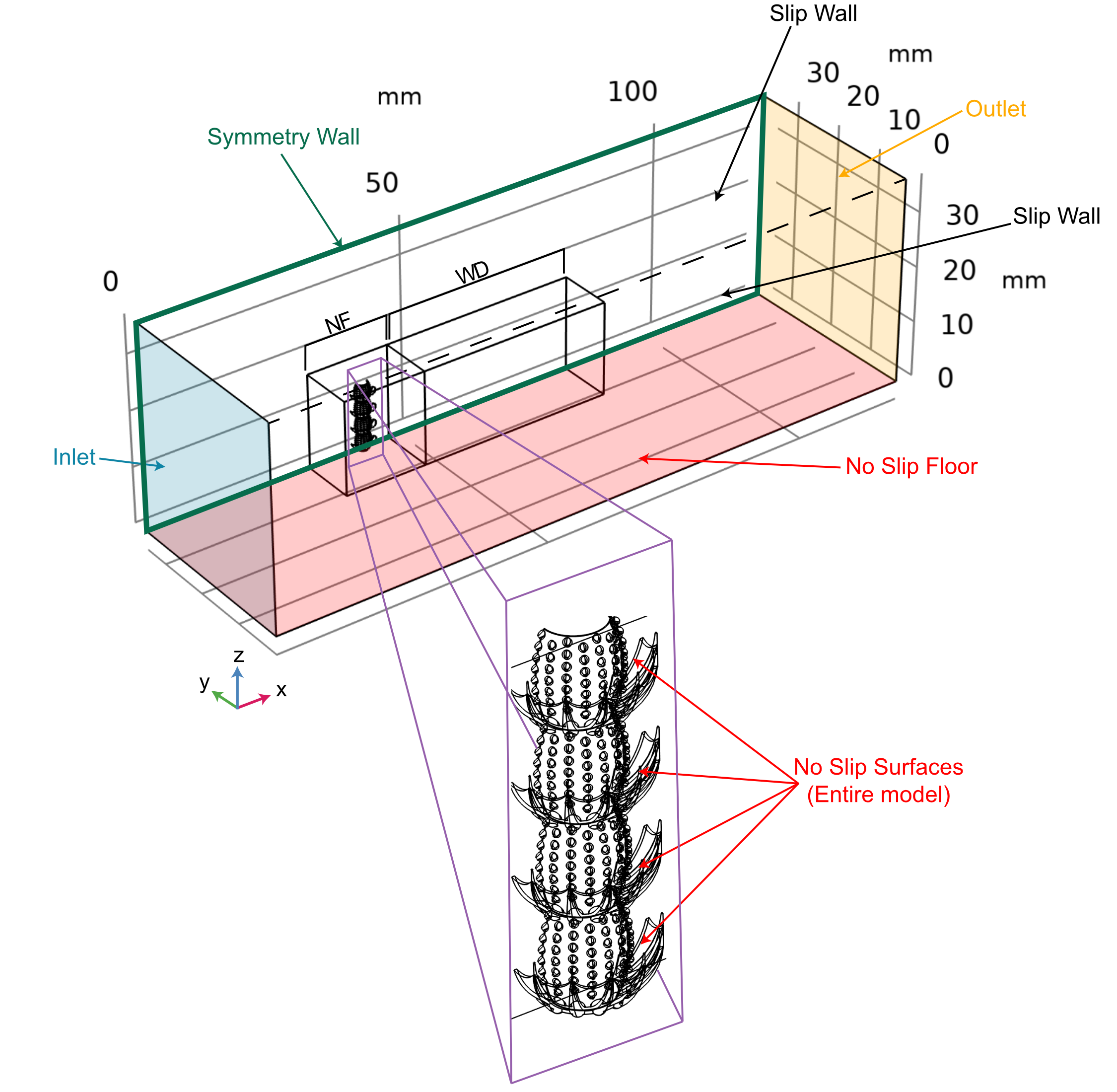

Supplement: S2 Fig — The symmetry wall is highlighted by a dark green outline. The near field (NF) and wake domain (WD) are also labelled. The purple lines zoom in onto the macroscopic model of Y. yukonensis. The flow domain floor and model are no slip surfaces. (TIF) [file pone.0347476.s005.tif]

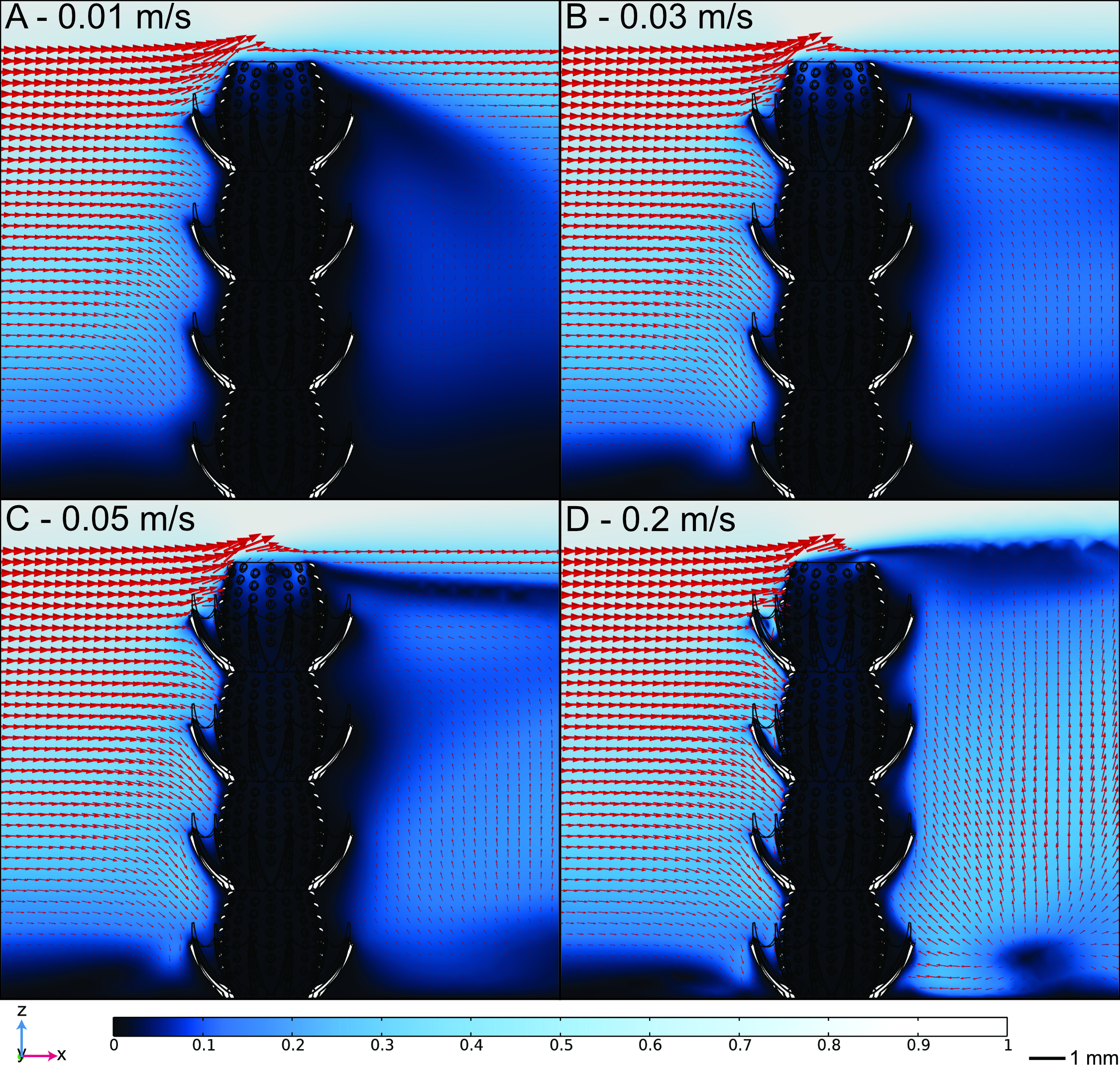

Supplement: S3 Fig — A) Velocity at 0.01 m s-1; B) Velocity at 0.03 m s-1; C) Velocity at 0.05 m s-1; D) Velocity at 0.2 m s-1. All arrows scaled by a factor of 1.5. (TIF) [file pone.0347476.s006.tif]

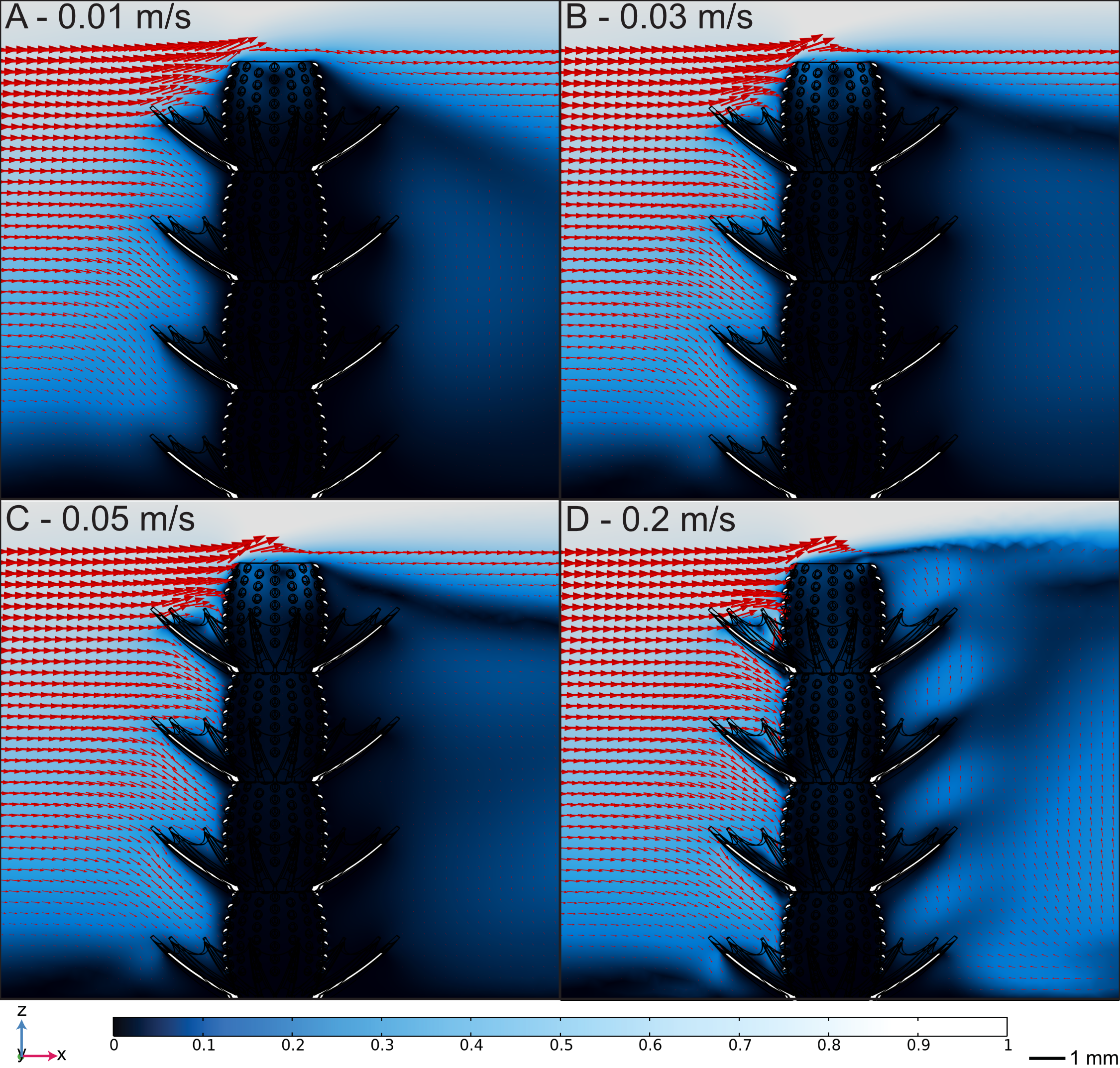

Supplement: S4 Fig — A) Velocity at 0.01 m s-1; B) Velocity at 0.03 m s-1; C) Velocity at 0.05 m s-1; D) Velocity at 0.2 m s-1. All arrows scaled by a factor of 1.5. (TIF) [file pone.0347476.s007.tif]

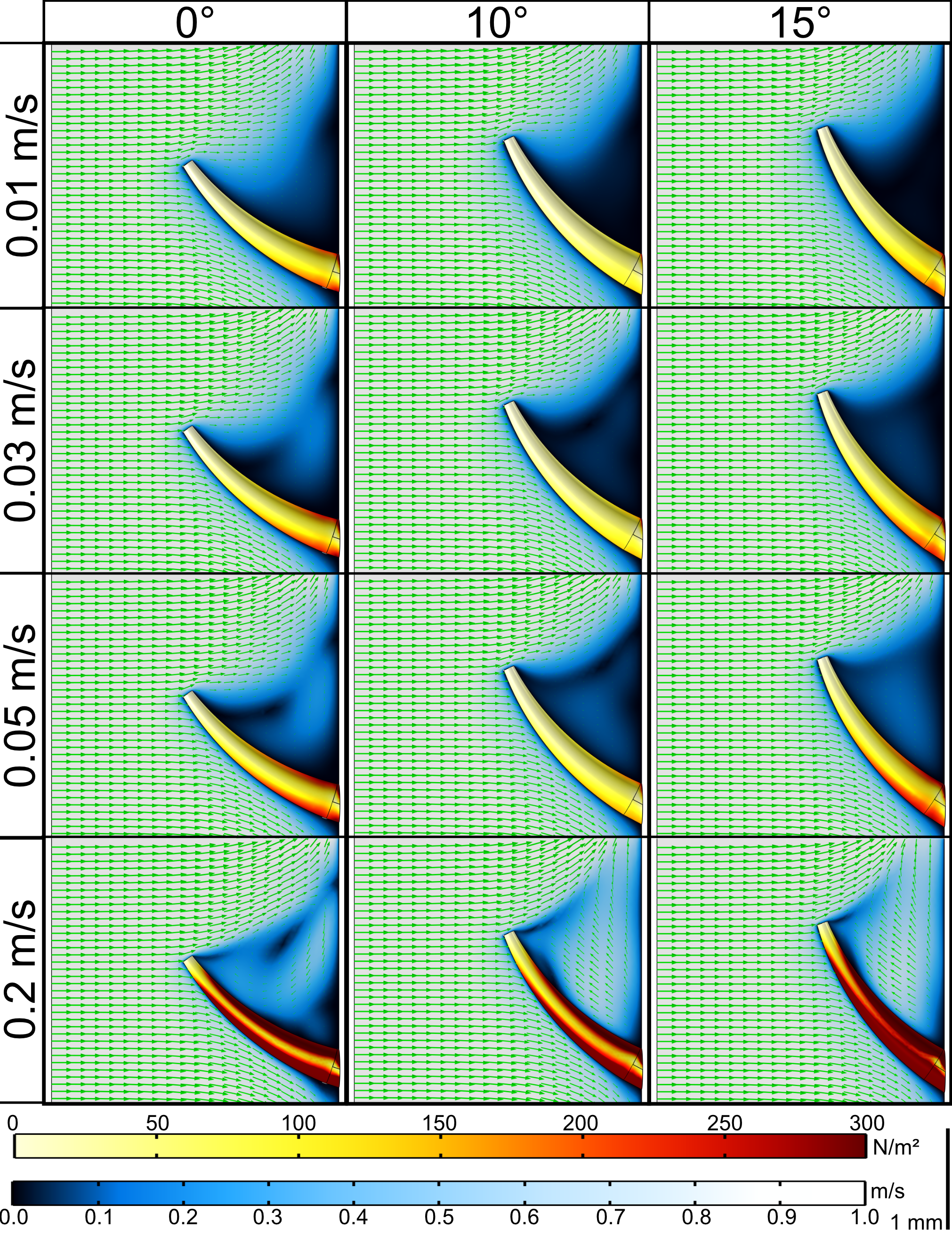

Supplement: S5 Fig — Green arrows represent the direction of the fluid’s movement with length corresponding to velocity magnitude (U). The von Mises Stress (N m-2) is visualized on the spine, while the normalized velocity is visualized around the flow domain. (TIF) [file pone.0347476.s008.tif]

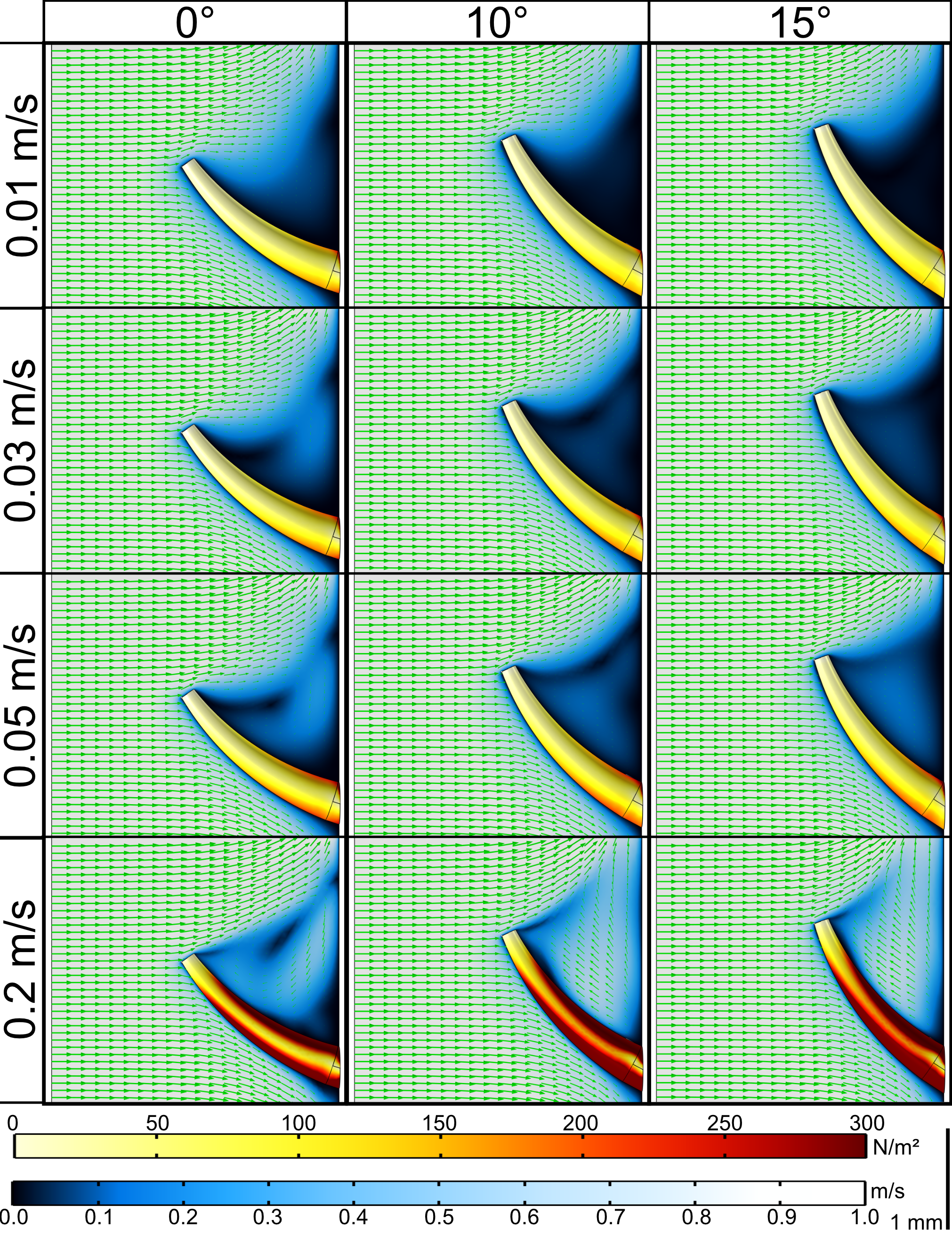

Supplement: S6 Fig — Green arrows represent the direction of the fluid’s movement with length corresponding to velocity magnitude (U). The von Mises Stress (N m-2) is visualized on the spine, while the normalized velocity is visualized around the flow domain. (TIF) [file pone.0347476.s009.tif]

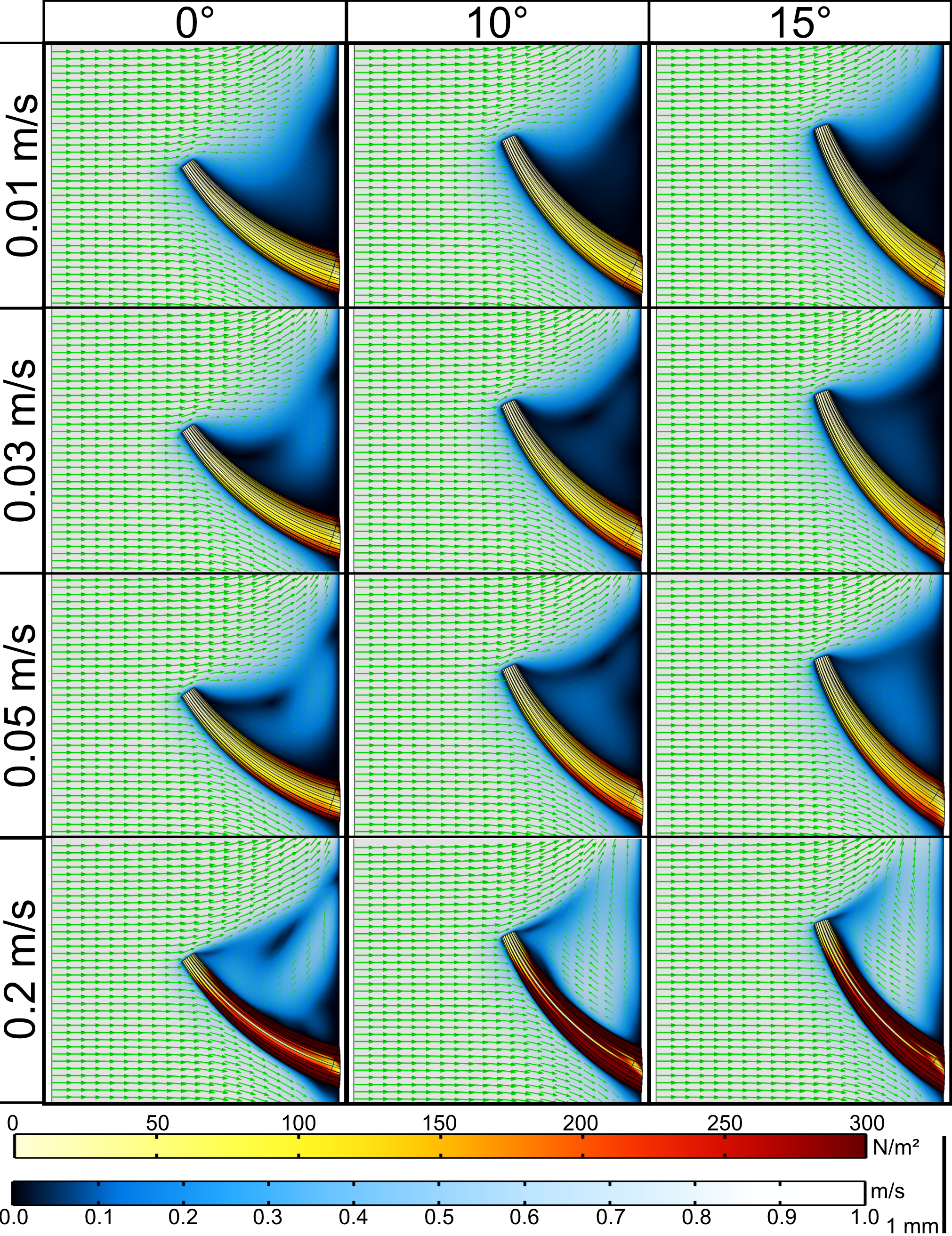

Supplement: S7 Fig — Green arrows represent the direction of the fluid’s movement with length corresponding to velocity magnitude (U). The von Mises Stress (N m-2) is visualized on the spine, while the normalized velocity is visualized around the flow domain. (TIF) [file pone.0347476.s010.tif]

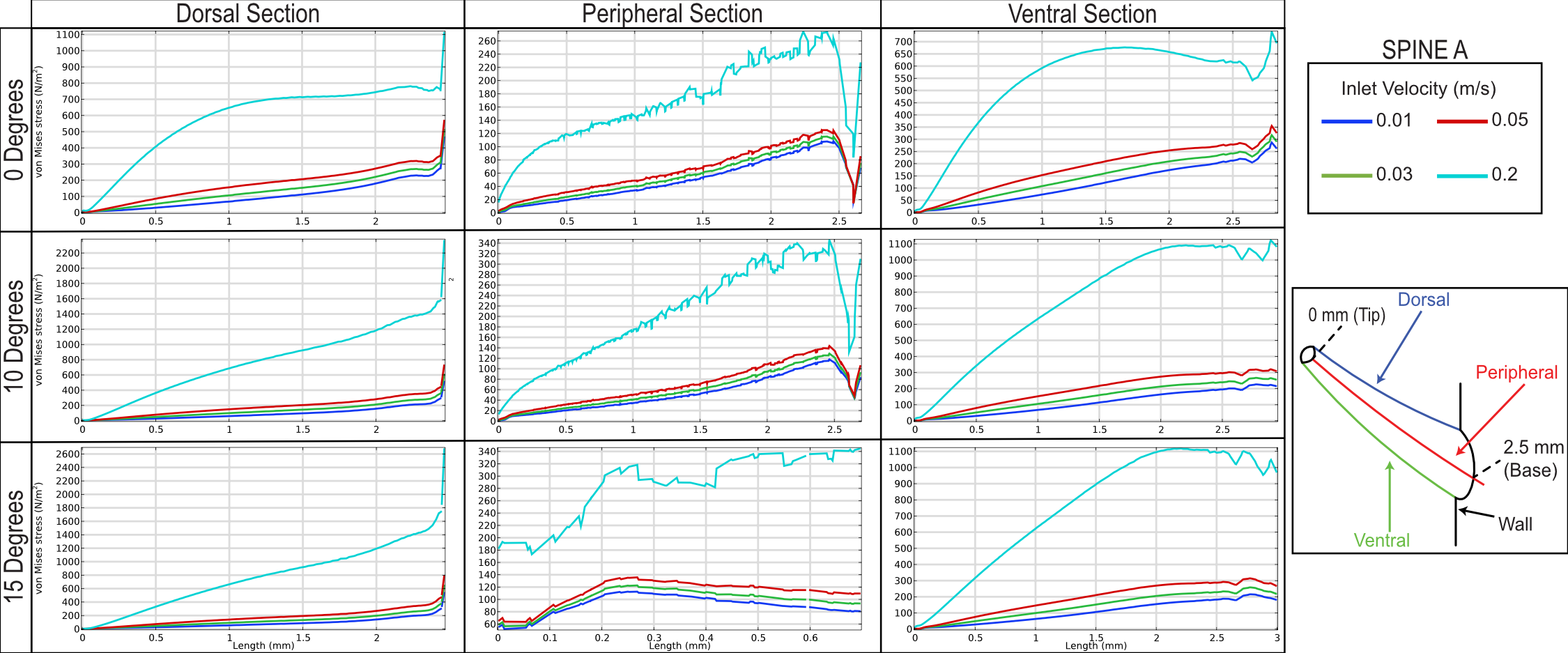

Supplement: S8 Fig — Diagram on the right shows the locations where the stress profiles were measured. The von Mises Stress is measured in N m-2. The coloured lines distinguish the four tested velocities at the three chosen angles and locations. (TIF) [file pone.0347476.s011.tif]

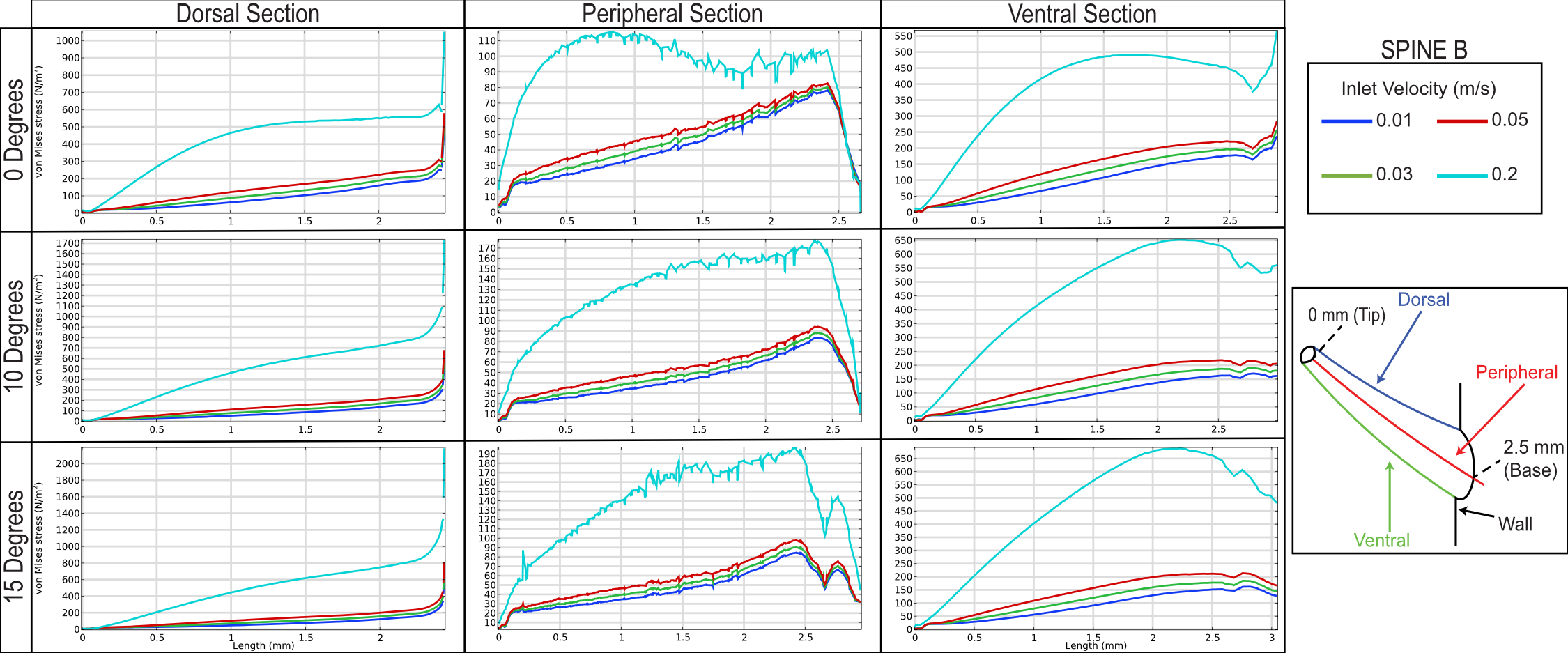

Supplement: S9 Fig — Diagram on the right shows the locations where the stress profiles were measured. The von Mises Stress is measured in N m-2. The coloured lines distinguish the four tested velocities at the three chosen angles and locations. (TIF) [file pone.0347476.s012.tif]

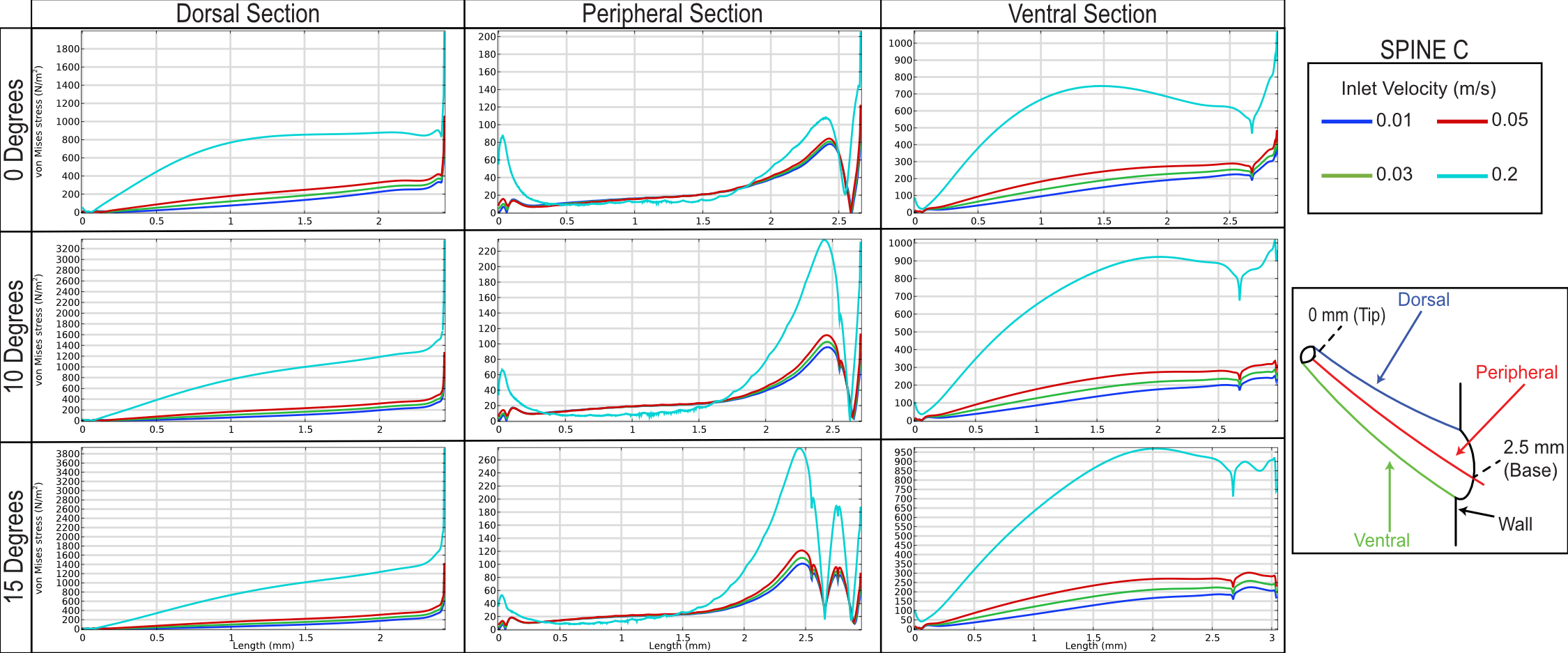

Supplement: S10 Fig — Diagram on the right shows the locations where the stress profiles were measured. The von Mises Stress is measured in N m-2. The coloured lines distinguish the four tested velocities at the three chosen angles and locations. (TIF) [file pone.0347476.s013.tif]
