# Supplementary material for: Functional morphology of the Cambrian archaeocyath sponge Yukonensis
Source: PLoS One. 2026 May 11;21(5):e0347476. doi: 10.1371/journal.pone.0347476 (PMC13160350; doi:10.1371/journal.pone.0347476)
Supplement: Attachment — Submitted filename: Response to Reviewer.docx [file pone.0347476.s015.docx]

**Journal Requirements:**

 1. When submitting your revision, we need you to address these additional requirements.

Please ensure that your manuscript meets PLOS ONE's style requirements, including those for file naming. The PLOS ONE style templates can be found at

https://journals.plos.org/plosone/s/file?id=wjVg/PLOSOne_formatting_sample_main_body.pdf and

https://journals.plos.org/plosone/s/file?id=ba62/PLOSOne_formatting_sample_title_authors_affiliations.pdf

2. Please update your submission to use the PLOS LaTeX template. The template and more information on our requirements for LaTeX submissions can be found at http://journals.plos.org/plosone/s/latex.

3. In your manuscript, please provide additional information regarding the specimens used in your study. Ensure that you have reported human remain specimen numbers and complete repository information, including museum name and geographic location.

If permits were required, please ensure that you have provided details for all permits that were obtained, including the full name of the issuing authority, and add the following statement:

'All necessary permits were obtained for the described study, which complied with all relevant regulations.'

If no permits were required, please include the following statement:

'No permits were required for the described study, which complied with all relevant regulations.'

CHANGES MADE: Since no permits were required for this study, this statement has been added in Lines 146 – 147.

For more information on PLOS One's requirements for paleontology and archeology research, see https://journals.plos.org/plosone/s/submission-guidelines#loc-paleontology-and-archaeology-research.

4. We note that the grant information you provided in the ‘Funding Information’ and ‘Financial Disclosure’ sections do not match.

When you resubmit, please ensure that you provide the correct grant numbers for the awards you received for your study in the ‘Funding Information’ section.

5. Thank you for stating in your Funding Statement:

BMG and ML were supported by the Dutch Research Council (NWO; grant number OCENW.M.21.031; https://www.nwo.nl/en/find-funding), and ML was supported by Natural Sciences and Engineering Research Council of Canada (2019-05405 ML; https://www.nserc-crsng.gc.ca/index_eng.asp). ZAQ was supported by the Natural Sciences and Engineering Research Council Undergraduate Student Research Award (https://www.nserc-crsng.gc.ca/students-etudiants/ug-pc/usra-brpc_eng.asp). Many of the techniques used in this research were developed thanks to joint funding from the US National Science Foundation (https://www.nsf.gov) and UK Natural Environment Research Council (NSF-NERC EAR-2007928; https://www.ukri.org/councils/nerc/). Sponsors had no role in influencing this study.

Please provide an amended statement that declares *all* the funding or sources of support (whether external or internal to your organization) received during this study, as detailed online in our guide for authors at http://journals.plos.org/plosone/s/submit-now.  Please also include the statement “There was no additional external funding received for this study.” in your updated Funding Statement.

Please include your amended Funding Statement within your cover letter. We will change the online submission form on your behalf.

6. Thank you for stating the following financial disclosure:

BMG and ML were supported by the Dutch Research Council (NWO; grant number OCENW.M.21.031; https://www.nwo.nl/en/find-funding), and ML was supported by Natural Sciences and Engineering Research Council of Canada (2019-05405 ML; https://www.nserc-crsng.gc.ca/index_eng.asp). ZAQ was supported by the Natural Sciences and Engineering Research Council Undergraduate Student Research Award (https://www.nserc-crsng.gc.ca/students-etudiants/ug-pc/usra-brpc_eng.asp). Many of the techniques used in this research were developed thanks to joint funding from the US National Science Foundation (https://www.nsf.gov) and UK Natural Environment Research Council (NSF-NERC EAR-2007928; https://www.ukri.org/councils/nerc/). Sponsors had no role in influencing this study.

Please state what role the funders took in the study.  If the funders had no role, please state: "The funders had no role in study design, data collection and analysis, decision to publish, or preparation of the manuscript."

If this statement is not correct you must amend it as needed.

Please include this amended Role of Funder statement in your cover letter; we will change the online submission form on your behalf.

7. In the online submission form, you indicated that your data will be submitted to a repository upon acceptance.  We strongly recommend all authors deposit their data before acceptance, as the process can be lengthy and hold up publication timelines. Please note that, though access restrictions are acceptable now, your entire minimal dataset will need to be made freely accessible if your manuscript is accepted for publication. This policy applies to all data except where public deposition would breach compliance with the protocol approved by your research ethics board. If you are unable to adhere to our open data policy, please kindly revise your statement to explain your reasoning and we will seek the editor's input on an exemption.

8. When completing the data availability statement of the submission form, you indicated that you will make your data available on acceptance. We strongly recommend all authors decide on a data sharing plan before acceptance, as the process can be lengthy and hold up publication timelines. Please note that, though access restrictions are acceptable now, your entire data will need to be made freely accessible if your manuscript is accepted for publication. This policy applies to all data except where public deposition would breach compliance with the protocol approved by your research ethics board. If you are unable to adhere to our open data policy, please kindly revise your statement to explain your reasoning and we will seek the editor's input on an exemption. Please be assured that, once you have provided your new statement, the assessment of your exemption will not hold up the peer review process.

9. We note that Figure 1 in your submission contain copyrighted images. All PLOS content is published under the Creative Commons Attribution License (CC BY 4.0), which means that the manuscript, images, and Supporting Information files will be freely available online, and any third party is permitted to access, download, copy, distribute, and use these materials in any way, even commercially, with proper attribution. For more information, see our copyright guidelines: http://journals.plos.org/plosone/s/licenses-and-copyright.

We require you to either present written permission from the copyright holder to publish these figures specifically under the CC BY 4.0 license, or remove the figures from your submission:

a. You may seek permission from the original copyright holder of Figure 1 to publish the content specifically under the CC BY 4.0 license.

We recommend that you contact the original copyright holder with the Content Permission Form (http://journals.plos.org/plosone/s/file?id=7c09/content-permission-form.pdf) and the following text:

“I request permission for the open-access journal PLOS ONE to publish XXX under the Creative Commons Attribution License (CCAL) CC BY 4.0 (http://creativecommons.org/licenses/by/4.0/). Please be aware that this license allows unrestricted use and distribution, even commercially, by third parties. Please reply and provide explicit written permission to publish XXX under a CC BY license and complete the attached form.”

Please upload the completed Content Permission Form or other proof of granted permissions as an "Other" file with your submission.

In the figure caption of the copyrighted figure, please include the following text: “Reprinted from [ref] under a CC BY license, with permission from [name of publisher], original copyright [original copyright year].”

CHANGES MADE: Permission has been granted and this statement has been included in the Figure 1 caption (Lines 89 – 91). A copy of proof of permission has been uploaded to the submission portal.

b. If you are unable to obtain permission from the original copyright holder to publish these figures under the CC BY 4.0 license or if the copyright holder’s requirements are incompatible with the CC BY 4.0 license, please either i) remove the figure or ii) supply a replacement figure that complies with the CC BY 4.0 license. Please check copyright information on all replacement figures and update the figure caption with source information. If applicable, please specify in the figure caption text when a figure is similar but not identical to the original image and is therefore for illustrative purposes only.

10. Please upload a new copy of Figures 4, 5, S8, S9, S10, S11, S12 and S13 as the detail is not clear. Please follow the link for more information:  <https://journals.plos.org/plosone/s/figures>

CHANGES MADE: Figures have been modified and re-uploaded to be clearer. All figures and supplementals follow the required formats of PLOS One. Changes are described below in the reviewer comments section.

11. If the reviewer comments include a recommendation to cite specific previously published works, please review and evaluate these publications to determine whether they are relevant and should be cited. There is no requirement to cite these works unless the editor has indicated otherwise. 

**REVIEWER COMMENTS**

5. Review Comments to the Author

Please use the space provided to explain your answers to the questions above. You may also include additional comments for the author, including concerns about dual publication, research ethics, or publication ethics. (Please upload your review as an attachment if it exceeds 20,000 characters)

**Reviewer #1:** This manuscript employs computational fluid dynamics modeling to test feeding strategies and structural stability in the enigmatic archaeocyath *Yukonensis yukonensis*. While the application of numerical modeling to this unique morphology is valuable, I have significant concerns regarding 1) the structure of the article, and 2) critical discrepancies between the actual fossil morphology and the geometry used for the simulation. I therefore suggest a major revision for the manuscript before publication. Of note, although I am familiar with archaeocyath, I am not a specialist in this specific taxon nor in functional morphology modeling, so I invite the authors to correct me if my interpretation of the model setup is mistaken.

1. It is difficult to follow the morphology of Yukonensis based on the current figures and text structure. Fig 1B, C does not clearly show the spine structure, yet the text describes model parameters before adequately visualizing the biological structure. As a reader, I found it confusing to reconcile the "wide" vs. "narrow" corolla descriptions (Fig 1A vs 1D) with the single species description. As a paleontologist, I believe it is necessary to fully describe the reconstructed biological structure of Yukonensis before introducing the modeling parameters, a step that is currently missing in the manuscript.

CHANGES MADE: We have added additional descriptions of the morphological terminology and forms of typical archaeocyaths earlier in the introduction to make the morphological descriptions of *Y. yukonensis* clearer by introducing them before discussing the models that we have constructed.

Lines 50 - 57: “In broad terms, archaeocyaths are characterized by relatively thick, porous, calcifying skeletons that range from cylindrical to conical and typically display a characteristic two-walled ‘cone-in-cone’ architecture [8]. The space between the walls is known as the intervallum and can contain a diverse array of calcium carbonate features that vertically and horizontally partition the skeleton of the archaeocyath [8]. Moreover, some archaeocyaths in the Order Monocyathida instead have a single wall and completely lack an intervallum [20]. Although these forms are often treated as the archetypal archaeocyath morphology, numerous taxa diverge markedly from this body plan [7,19–21].”

CHANGES MADE: We appreciate the reviewer’s feedback on our manuscript’s Introduction and Figure 1. Figure 1 has been modified in several ways to increase clarity. First, Fig 1B now shows a remodelled *Y. yukonensis* that has spines on the thorny corolla. Additionally, to reduce confusion about the wide and narrow variants, a model of the narrow variant has been added to Fig 1C. Similarly, the cross-section schematic of *Y. yukonensis* (Fig 1D, previously Fig 1C) also has spines included. In Fig 1C, an error where VII and VIII pointed to the same feature has also been fixed. Fig 1E is now the 2D schematic showing how the model was created along with measurements of the features. The final two panel figures (now Fig 1F and G) remain unchanged. This modified figure in combination with the glossary table (Table 1) provides further clarity on the organism and its morphology.

Lines (77-91): “Fig 1. Fossil specimens and reconstructions of *Yukonensis yukonensis* from the Mackenzie Mountains, Yukon, Canada. A) Longitudinal section (from Handfield [22], Plate 1, Fig 4). B) Two chambers of a reconstructed 3D *Y. yukonensis* model with narrow thorny corolla. C) Two chambers of a wide-angled thorny corolla *Y. yukonensis* reconstruction. D) Cross-sectional diagram of a single chamber. The red colour indicates internal features that were not modelled (See Materials and Methods). The labels are as follows: I) Osculum, II) Rods, III) Synapticulae, IV) Outer wall pore, V i) Spine, V ii) Connecting membrane, V) Thorny corolla, VI) T-shaped ridge (enlarged), VII) Inner wall pore , VIII) Central cavity, IX) Tumuli, X) Inner wall, XI) Outer wall, XII) Vesicle, XIII) Intervallum. E) Two-dimensional view of the thorny corolla with labelled measurements of key features. The narrow thorny corolla is labelled with ‘n’, and the wide thorny corolla variant is labelled with ‘w’, F-G) Cross-section images from Handfield and McKinney [21] displaying the intervallum, the open central cavity between chambers (E; Plate 1, Fig 1) and the aporous horizontal vesicle membrane (red arrow - F; Plate 2, Fig 2). Sections A, E, and F reprinted from [21,22] under a CC BY license, with permission from Sedimentary Geology (SEPM), original copyright [1967, 1975].”

2. There are critical discrepancies between the organism described in the text/images and the 3D geometry used for the simulations, which likely compromise the validity of the flow model results.

- The characteristic double-walled structure of archaeocyaths is clearly visible in the photomicrographs (Fig 1E, F) and the diagram (Fig 1C). However, the model (Fig 1B) lacks an inner wall entirely. The authors state this was done to "focus solely on the external structure" (lines 183-184), but for a sponge-grade organism, flow is dictated by the pressure gradient across the intervallum (Savarese, 1992). Removing the inner wall would fundamentally alter the internal flow resistance. This is particularly important given that the authors are interpreting the organism as an active filter feeder.

CHANGES MADE: Due to computational limitations, we were unable to change the model by adding the radial elements and inner wall. However, we have addressed the reviewer’s concerns regarding the conclusions of our paper on the active feeding mechanisms of *Yukonensis*. We have focussed our discussion on the vertical sectioning of flow and the potential functional roles of the thorny corolla, spines, and T-ridges. We have listed all our changes regarding feeding to the last comment of this review.

- The authors model the complex thorny corolla as "two-dimensional planes with no thickness" (Lines 182-183). However, the text describes the corolla as an "inverted umbrella" with distinct spines, and the authors note spines are up to 0.5 mm in diameter (Line 158, Fig. 1E). Modeling such large structures with zero thickness treats the corolla as a smooth baffle. In reality, thick spines would create surface rugosity and turbulence, potentially mixing flow into the pores, a mechanism completely lost in the simplified model.

CHANGES MADE: We greatly appreciate the reviewer’s insight on the lack of surface roughness on our original model. To fix this, we have remodelled our *Yukonensis* model so that the thorny corolla have the curvature of the spines present. Furthermore, we have changed the boundary conditions of our set up, so the thorny corolla remain 3D rather than internal boundaries, which we prescribed before. Overall, this physically adds macroscale surface topography to the thorny corolla, meaning their potential mechanism is no longer lost in our model and simulations. Mentions of the thorny corolla as two-dimensional planes have been removed from Figure 1 and the Materials and Methods section of our manuscript. Our new Figure 1B, C, and D panels reflect this change.

- While the text acknowledges "curved, hollow spines" (and Fig 1E shows a clavate/club-like shape), the methodology simplifies them into straight cones. Given that the study aims to test flow redirection, straightening a curved feature removes the very trait (curvature) that would most likely redirect fluid.

CHANGES MADE: We thank the reviewer for the comment on the spine model and curvature. We have replaced the straight, cone-like spine with a curved model. This spine better reflects the descriptions of *Yukonensis* in the literature. We have kept the overall design of testing at three distinct angles; however, the angles have changed from 30, 60, 90 degrees to 0, 10, 15 degrees from the outer wall. Additionally, the spine has been modelled as a hollow structure with a thickness of 0.05 mm to better reflect the morphology of the spines of *Yukonensis*. These new angles and the results of these new models are described in the Results section. All relevant figures and supplemental documents have been modified to reflect the change in morphology (Fig 4; S1Fig, S5-10Figs).

Lines 180 – 191: “We used FSI to assess fluid-derived stress distributions along the length of three hypothesized spine models. All spine models are approximately 2.5 mm in length depending on the angle. Spine A and Spine B are both curved cones with diameters (at the proximal attachment point) of 0.50 mm and 0.556 mm [21], respectively. Spine C includes ridges, with an outer ridge diameter of 0.556 mm and inner ridge diameter of 0.50 mm, corresponding to Spines B’s and A’s diameters, respectively. All spines are hollow in cross-section with a shell thickness of 0.05 mm. Spine B allowed for testing if differences in our results between Spine A and Spine C were due to the presence of the ridges and not merely the larger diameter in those regions. Since the angles at which the spines protruded out of living *Y. yukonensis* are unknown, three separate angles were chosen to investigate how this angle could affect stress based on fossil specimen photographs: 0º, 10º, and 15º to the body wall.”

- The full-body model uses only four chambers, whereas Fig. 1A shows at least six. The flow results (Fig 2) show significant differences between the top and bottom chambers. The authors should justify why four chambers are sufficient to represent a mature organism or conduct a sensitivity test with more chambers to ensure the observed "dead zone" is not simply an inlet/outlet boundary artifact.

CHANGES MADE: We acknowledge the reviewer’s concerns on the lack of justification for the number of chambers. We have included additional information regarding the morphology of *Yukonensis* chambers in the Introduction. This addition establishes that eighteen is the known maximum number of chambers and that most specimens show five or fewer chambers. We later justify this in the Materials and Methods.

Lines 62 – 63: “The largest known specimen of *Y. yukonensis* shows that it developed a maximum at least eighteen chambers, while most specimens have five or fewer [21].”

CHANGES MADE: These justifications below are made in the Materials and Methods.

Lines 140 – 142: “Note that the macroscopic model was constructed with four chambers which falls in range of most preserved *Y. yukonensis* specimens [22]. This lower chamber count was necessary to due to computational limitations, but it still permitted reconstructing flow across its chambers.”

Lines 211 – 214: “The macroscopic model of *Y. yukonensis* consists of four stacked porous chambers, each with a height of 3 mm cumulating to a total model height of 12 mm. A model with a total of four chambers was created based on most *Y. yukonensis* specimens preserving five or fewer chambers [22] and to reduce the number of meshing elements due to computational constraints.”

CHANGES MADE: The reviewer also raised concerns that the ‘dead zones’ in our results may be a result of inlet/outlet boundary artifacts. We believe it is unlikely that these artifacts are present and influencing our results due to our flow domain set-up. We aim to limit artifacts as much as possible by placing the model at a one-third distance from the inlet boundary condition. Given the millimetre scale of the model, this provides ample space to prevent interference from the inlet, outlet, and surrounding slip walls. We have clarified this further in the Materials and Methods.

Lines 222 – 224: “Models were placed in a hexahedral flow domain of dimensions 120 mm *x* 70 mm *x* 40 mm (W *x* D *x* H) (S5 Fig). Each model was placed in the center of the lower surface at one-third the streamwise distance from the inlet (40 mm) to prevent numerical anomalies arising from boundary conditions [38].”

- The authors conclude Yukonensis was an active feeder because the passive model resulted in stagnant flow. However, as discussed above, because the model oversimplifies the morphology of the organism, this result may be an artifact of the geometry rather than the biology. The conclusion should be softened to state that passive flow is inefficient under these specific geometric constraints.

CHANGES MADE: Thank you for the comment and suggestion regarding the conclusions in our manuscript. We have made several major modifications to shift the focus of our paper from feeding mechanisms as detailed below:

CHANGES MADE: In our Materials and Methods, we state that the internal components of the organism are not modelled due to computational restraints introduced from increasing complexity.

Lines 212 – 216: “A model with a total of four chambers was created based on most *Y. yukonensis* specimens preserving five or fewer chambers [22] and to reduce the number of meshing elements due to computational constraints. Similarly, only the outer wall of the organism was modelled to focus solely on the external structure of *Y. yukonensis*.”

CHANGES MADE: To ensure clarity on what our model tests and what it does not, we have removed internal labels from Figure 2 (such as internal circulation and flow out the osculum). Instead, our new labels and results focus solely on the thorny corolla and the outer wall. This keeps our Results section grounded to solely what our model can test.

Lines 252 – 255: “Fig 2. CFD results for U = 0.2 m s^-1^ inlet flow on the Y. yukonensis narrow macroscopic reconstruction. Colour corresponds to the velocity normalized to the inlet. A: Viscous sublayer; B: Downstream wake; C: Acceleration of fluid entering upper thorny corolla; D: Reduced circulation within bottom-most thorny corolla; E: Downstream deceleration around thorny corolla*.*”

CHANGES MADE: In the Discussion section, our analysis remains focused on the external fluid movement and using these results based on our reconstruction.

Lines 350 – 353: “Specifically, we find that the morphology of the thorny corolla impeded fluid flow from entering the tumuli, thus making it an unlikely adaptation to facilitate passive suspension feeding. Likewise, we find that the morphology of T-shaped ridges neither re-direct flow towards tumuli, nor do they reduce mechanical stress in the spines under a range of realistic flow regimes.”

CHANGES MADE: Later in the Discussion, we use the results from our model to make inferences about feeding processes and the influence of internal structures. Following the Reviewer’s advice, we emphasize that our discussion on feeding and internal components is based on the results given by a model that solely contains external surface.

Lines 361 – 368: “Based on our external reconstruction, the results (Figs 2E, 2G, and 3) show tentative support for an active filter-feeding interpretation for *Y. yukonensis*. For passive feeding to be feasible, ambient flow must efficiently circulate into the central cavity via the tumuli and subsequently exit the organism, presumably through the osculum [29]. In contrast, active feeding involves the expenditure of energy such as the beating motion of flagellated units to pump water in and out of a sponge. In our simulations, we demonstrate that the presence of a thorny corolla accelerated flow along the thorny corolla’s inside curvature, but advected flow away from the organism’s tumuli (Fig 2C, D).”

Lines 377 – 386: “Although computational constraints preclude detailed analysis of flow within the internal chambers, our external results provide a basis for informed assumptions. In fossil specimens, the intervallum of *Yukonensis* includes a localized lattice of cylindrical rods cross‑linked by synapticulae (i.e., short connecting bars) that occurs only at the uppermost portion of each chamber (Fig 1D). Because this framework does not extend continuously through the chamber, it likely had little influence on internal flow (Fig 1). The internal calcite rods and synapticulae may have provided structural support by bracing the intervallum and mechanically linking the inner and outer walls. Furthermore, they may have served as attachment sites for soft tissues (i.e. choanocytes) that have not yet been preserved in any archaeocyath fossil [19], and precludes their incorporation into the models.”

CHANGES MADE: In the Discussion, Conclusion, and Abstract, conclusive statements about the feeding ecology of *Yukonensis* have been removed based on the recommendation of the reviewer to reduce overinterpretation of our results and soften the conclusion.

Lines 501 – 503: “The advection of fluid around the tumuli and downstream within the thorny corolla indicates inadequate nutrient delivery inside the organism, implying a case for active feeding based on our reconstructed model.”

ADDITIONAL CHANGES:

We are very grateful for the in-depth feedback provided by the reviewer on our manuscript. In addition to the comments made by the reviewer, we have made several additions to further improve our manuscript that are detailed below:

- Throughout the manuscript, grammar and phrasing have been improved for clarity
- Manuscript and supplemental figures have been modified by normalizing the velocity to the inlet, which allows for better comparisons between different tested velocities
- Figure 3 has been modified to make panels A and B larger to easily compare the two models
- Figure 4 now only displays visualizations for Spine C (the ridged variant). Separate panels have been added to show detailed views of the von Mises stress on the tips and bases at the most extreme velocities and angles (Lines: 298 – 307).
- The Discussion section contains additional interpretation on the purpose of vesicles in *Y. yukonensis* and their potential impacts on its functional morphology (Lines: 404 – 426).
- In the Discussion, an additional paragraph has been added comparing the hollow spines of *Yukonensis* to other potential analogues (Lines: 455 – 463).
- Supplemental figures 5, 6, and 7 have been improved in their clarity by moving scales, which allows the spine plots to be enlarged and easier to view.

3. Minor Comments
- Line 179 lists the Young’s Modulus of the spine as 8897 GPa. The Young’s Modulus of calcite is typically ~70–80 GPa. Is this a typo for 88.97 GPa?

CHANGES MADE: As the reviewer suggested, this was a typographic error in our manuscript, and this has been corrected to the value used in our FSI simulations, 88.297 GPa.

Lines 207 – 208: (*r_s_*) = 2711 kg m^-3^, Young’s Modulus (*E*) = 88.197 GPa, and Poisson’s Ratio (*n*) = 0.33.

- Table 2 states the distance between spines is 4.8–6.6 mm, with 13 to 16 spines. This seems disproportionately large compared to the chamber size and differs significantly from the dimensions used in the model (1.105–1.358 mm). Please verify if these dimensions are correct relative to the fossil specimens.

CHANGES MADE: As suggested by the reviewer, these spine distance values were verified. We agree that these measurements disproportionately large compared to the organism. Based on Handfield (1967), these values report the lateral measurement between spine tips, rather than the distance between adjacent spines. We have changed the listed measurements in the table to match our model (Line 152).
